# Breaching the Blood-Brain Barrier: Evolving Strategies for Central Nervous System Disease in Adult Acute Lymphoblastic Leukemia

**DOI:** 10.1007/s11899-026-00782-5

**Published:** 2026-07-13

**Authors:** Liesl S. Eibschutz, Samuel Crow, Caroline Tatum, Daniel Reed, Michael Keng

**Affiliations:** University of Virginia Comprehensive Cancer Center, 1240 Lee St., Charlottesville, VA 22903 USA

**Keywords:** Acute lymphoblastic leukemia, Central nervous system, Intrathecal chemotherapy, CAR T-cell therapy, Blinatumomab, Inotuzumab ozogamicin, CNS prophylaxis

## Abstract

**Purpose of Review:**

Central nervous system (CNS) involvement in adult acute lymphoblastic leukemia (ALL) remains a critical determinant of treatment failure and long-term survival. This review provides a comprehensive, evidence-based framework for the diagnosis, risk stratification, prophylaxis, and treatment of CNS disease in adult ALL, with emphasis on the evolving challenges introduced by immunotherapy-based and chemotherapy-sparing regimens and presents our institutional approach to CNS-directed therapy.

**Recent Findings:**

Modern CNS prophylaxis combining intrathecal chemotherapy with CNS-penetrating systemic agents has dramatically reduced CNS relapse rates, yet diagnostic and therapeutic limitations persist. The increasing use of immunotherapies, such as blinatumomab and inotuzumab ozogamicin, has improved systemic disease control, while CNS relapse is increasingly recognized in this context, particularly among heavily pretreated patients. This pattern likely reflects multiple factors, including limited CNS penetration, improved disease control that unmasks previously subclinical CNS involvement, and the high-risk biology of relapsed/refractory disease. In contrast, CD19-directed CAR T-cell therapies have demonstrated meaningful activity in CNS disease, while investigational strategies targeting leukemic trafficking pathways and IL-15 signaling offer additional preclinical promise.

**Summary:**

As adult ALL treatment has shifted toward chemotherapy-sparing regimens, new concerns have emerged regarding disease control within the CNS compartment. Durable remission will require integration of rigorous intrathecal prophylaxis, risk-adapted systemic therapy, and novel agents capable of penetrating the CNS microenvironment into every treatment algorithm.

## Introduction

Central nervous system (CNS) involvement remains a critical challenge in adult acute lymphoblastic leukemia (ALL), as the blood–brain barrier (BBB) limits the penetration of systemic therapies, enabling leukemic persistence. Without prophylaxis, over half of patients historically developed CNS relapse, a risk that modern regimens combining intrathecal chemotherapy with CNS-penetrant systemic agents have reduced to below 10% [1].

However, the therapeutic landscape of adult ALL is undergoing a fundamental shift. The emergence of immunotherapies and targeted agents, such as bispecific T-cell engagers (BiTE), antibody–drug conjugates, and chimeric antigen receptor (CAR) T-cell therapies, has transformed systemic disease control and enabled chemotherapy-sparing approaches. As these agents increasingly replace or reduce the use of CNS-penetrant systemic chemotherapy, questions have arisen regarding their ability to adequately address sanctuary-site disease, given that most have limited penetration across the BBB. Whether this translates into a meaningful increase in CNS relapse risk remains an area of active investigation, and current data have not established a definitive causal link. Nonetheless, the evolving treatment paradigm underscores the importance of re-evaluating CNS-directed strategies, with greater emphasis on intrathecal prophylaxis and integration of novel agents capable of overcoming the BBB. In this review, we summarize current understanding of CNS disease in adult ALL, focusing on diagnosis, treatment, and emerging approaches, and outline a practical, evidence-based framework for CNS-directed management.

## Diagnosis

The clinical presentation of CNS disease in ALL is highly variable, ranging from subtle, nonspecific symptoms to overt neurologic dysfunction [2]. Patients may display headache or altered mental status, while focal findings can include cranial neuropathies, seizures, papilledema, or signs of spinal cord involvement such as weakness, sensory deficits, or gait disturbance [3]. However, many patients are asymptomatic at presentation, making clinical assessment alone unreliable for CNS detection [3]. Definitive diagnosis requires identification of leukemic blasts in the CSF via lumbar puncture (LP) [4]. The NCCN guidelines recommend that the timing of the initial lumbar puncture align with the chosen treatment regimen, typically with concurrent intrathecal therapy [5]. Neuroimaging is not required in asymptomatic patients, but should precede lumbar puncture in those with neurologic symptoms to exclude mass lesions or elevated intracranial pressure [6].

Gadolinium-enhanced magnetic resonance imaging (MRI) is the preferred imaging modality when CNS disease is suspected, with superior sensitivity for detecting leptomeningeal and parenchymal involvement [7]. Notably, a normal MRI does not rule out CNS involvement and should not be used as justification to forgo lumbar puncture [3]. Neuroimaging is most useful when leptomeningeal involvement is suspected, but cytology is negative or inadequate, when alternative diagnoses are considered, or when baseline imaging is needed for treatment planning, particularly if cranial irradiation is being considered [8].

CNS involvement is classified using a standardized CSF-based system (CNS-1 to CNS-3), which guides risk stratification and treatment decisions (Table 1) [9]. Traumatic lumbar punctures require interpretation using established correction algorithms to distinguish true CNS involvement from peripheral blood contamination.

**Table 1:** CSF Classification System for CNS Disease in ALL

CNS Status	CSF Criteria	Blast Presence	Clinical Criteria
CNS-1^a^	No blasts on cytospin	Absent	No clinical or radiological evidence of CNS involvement
CNS-2^b^	< 5 WBCs/µL on a non-traumatic LP; OR < 5 WBCs/µL with blasts on a traumatic LP not meeting CNS-3 criteria by the Steinherz/Bleyer algorithm	Present on cytospin	—
CNS-3^c^	≥ 5 WBCs/µL on a non-traumatic LP with blasts; OR a traumatic LP meeting the Steinherz/Bleyer algorithm criteria	Present on cytospin	OR: cranial nerve palsy; OR cerebral mass on neuroimaging — regardless of CSF WBC count

*Abbreviations*: *CNS* Central nervous system, *CSF* Cerebrospinal fluid, *LP* Lumbar puncture, *WBC* White blood cell

^a^CNS-1 is defined by the absence of detectable leukemic blasts on cytospin examination of a cytocentrifuged CSF sample and no clinical or radiological evidence of CNS involvement

^b^CNS-2 is an intermediate-risk category defined by the presence of leukemic blasts on cytospin in a CSF sample containing fewer than 5 WBCs/µL

^c^CNS-3 is defined in a non-traumatic LP as the presence of ≥ 5 WBCs/µL with identifiable leukemic blasts, or independently by the presence of a cerebral mass on neuroimaging, or a cranial nerve palsy with leukemic cells in the CSF, regardless of WBC count

Despite conventional cytology’s role as the diagnostic gold standard, it has well-recognized limitations. While specificity exceeds 95%, sensitivity is less than 50%, due to poor CSF cellularity and the difficulty of reliably distinguishing leukemic blasts from benign reactive cells, often necessitating multiple lumbar punctures [3]. Traumatic LPs pose an additional challenge, as they may introduce peripheral blasts into the CSF and have been associated with increased CNS relapse risk [10]. Retrospective studies have demonstrated a shorter relapse-free survival in patients with blast-positive traumatic LPs [10]. While flow cytometry offers significantly improved sensitivity compared to conventional cytology, feasibility may be limited by requirements for adequate CSF volume and timely processing [11]. Moreover, prospective validation in adults is still needed [12].

## Risk Factors

Several patient and disease-related factors increase the risk of CNS involvement and relapse in ALL. T-cell immunophenotype is a consistently recognized risk factor, with CNS relapse occurring at nearly twice the rate compared to the B-cell phenotype [13]. Hyperleukocytosis at presentation, defined as a WBC count exceeding 50 × 10⁹/L, is another well-established predictor of CNS disease [13]. Older age may contribute to increased CNS relapse risk through the higher rate of unfavorable comorbidities at baseline, reduced tolerance of CNS-directed therapy, and the need for dose modifications [14]. Elevated serum lactate dehydrogenase (LDH) has also been linked to CNS recurrence; Sancho et al. reported LDH > 1000 U/L as the only factor significantly associated with CNS relapse risk in adult ALL patients who did not receive cranial irradiation for CNS prophylaxis [15]. Additional cytogenetic and molecular risk factors include KMT2A rearrangement and mature B-ALL phenotype [16].

Philadelphia chromosome positivity (Ph+) is another important risk factor, accounting for approximately 20–25% of adult ALL cases, with an incidence that increases with age [17]. The hallmark of this subtype is the BCR::ABL fusion gene, arising from a translocation between chromosomes 9 and 22, which encodes a constitutively active tyrosine kinase oncoprotein that drives leukemic proliferation [18]. Ph + ALL is linked to a higher likelihood of CNS involvement and is often associated with aggressive disease progression [17].

## Components of CNS Prophylaxis

IT chemotherapy, administered via LP, remains the backbone of CNS prophylaxis. Common approaches include single-agent methotrexate, single-agent cytarabine, and triple intrathecal therapy (TIT), which combines methotrexate, cytarabine, and a corticosteroid [5]. The number of treatments varies by protocol, typically ranging from 8 to 17 doses [19]. Head-to-head comparisons among these regimens in adults are lacking, and neither the NCCN nor the American Society of Hematology (ASH) guidelines specify a preference [5, 20].

### Liposomal Cytarabine

Although liposomal cytarabine prolongs CSF exposure, randomized studies demonstrated substantially higher neurotoxicity than triple intrathecal therapy, limiting routine use [21, 22].

### Corticosteroid Selection

Dexamethasone achieves superior CNS penetration and is incorporated into most contemporary adult ALL regimens, although randomized adult data have not demonstrated a clear CNS-specific advantage over prednisolone [23–25]. In the IT setting, hydrocortisone, rather than dexamethasone, is the corticosteroid most commonly used as part of triple intrathecal therapy [26].

### Pegylated Asparaginase

Pegylated asparaginase (pegaspargase) contributes indirectly to CNS control and remains incorporated into many pediatric-inspired regimens, although its independent effect on CNS relapse prevention is uncertain [5, 27].

### Cranial Irradiation

Cranial irradiation, historically the standard for CNS prophylaxis, has been largely abandoned due to long-term neurocognitive toxicity, endocrinopathy, and secondary malignancies [28]. It is now reserved for overt CNS disease (CNS-3) at diagnosis persisting after induction, or IT-refractory disease. The NCCN recommends 18 Gy in 1.8–2.0 Gy fractions, with the field encompassing the entire brain, posterior half of the globe, and extending inferiorly to C2 [5].

## Treatment of Overt CNS Disease

### CNS Disease at Diagnosis

Overt CNS leukemia at diagnosis, defined as CNS-3 status, is identified in approximately 5%–10% of adult ALL patients [29]. Although relatively uncommon, its detection mandates a distinct therapeutic approach that integrates intensified CNS-directed therapy with standard systemic treatment [30]. The recommended CNS-directed schedule is twice-weekly TIT until CSF clearance of blasts, followed by weekly administration for 4–8 weeks, then every other week for an additional 4–8 doses, and subsequently monthly, for a total of 16–20 TIT administrations [19]. This intensified IT schedule is given with standard induction and consolidation chemotherapy, tailored to the patient’s risk level (low, intermediate, or high) [31]. Notably, when CNS disease at diagnosis is treated appropriately with this intensified approach, survival outcomes are comparable to those of CNS-negative patients, though it has been associated with a higher risk of subsequent CNS relapse in large trials [32]. For CNS leukemia persisting after induction, the NCCN guidelines recommend cranial irradiation as mentioned above [5].

### CNS Relapse

The treatment approach for CNS relapse requires a multimodal strategy that addresses both the CNS compartment and the systemic disease simultaneously. Intensive systemic chemotherapy with CNS-penetrating agents (high-dose methotrexate and high-dose cytarabine) is combined with intensified TIT using the same twice-weekly-to-monthly schedule described for de novo CNS disease, with cranial or craniospinal irradiation reserved for patients with IT-refractory disease [5]. This principle of concurrent systemic therapy is particularly critical in the setting of isolated CNS relapse, as extramedullary relapse is rarely truly isolated, and without adequate systemic therapy, subsequent bone marrow relapse is highly likely [3]. Allogeneic hematopoietic stem cell transplantation (HSCT) should be pursued for patients achieving complete response (CR) after CNS relapse when possible. In the Campus ALL study, patients who achieved CR and subsequently underwent allogeneic transplant had significantly improved overall survival compared to non-transplanted patients (*p* = 0.012) [13]. CAR T-cell therapy has also demonstrated meaningful activity in CNS relapse, as discussed below, and represents an increasingly important therapeutic option.

### Post-HSCT CNS Relapse

CNS relapse after allogeneic HSCT is a distinct clinical entity with unique therapeutic challenges. It occurs in an immunocompromised patient who is already exposed to intensive conditioning and is often less able to tolerate further cytotoxic therapy, requiring a carefully balanced multimodal approach. Management generally mirrors treatment of CNS relapse in non-transplant patients and includes intensified intrathecal therapy, CNS-penetrating systemic therapy, and selected use of radiation [5, 33]. Donor lymphocyte infusion (DLI) and second allogeneic transplantation may be considered in carefully selected patients, although outcomes remain poor [34–36].

## Novel and Emerging Therapies for CNS Disease

A central challenge of the modern treatment era is that immunotherapy agents with limited CNS penetration, particularly blinatumomab and inotuzumab ozogamicin, are increasingly replacing CNS-penetrating chemotherapy in frontline and salvage regimens. Although these agents have transformed disease control, sanctuary-site relapses, including CNS relapse, are increasingly recognized in this context, particularly among heavily pretreated patients [37]. This observation likely reflects multiple factors, including improved disease control that unmasks previously subclinical CNS involvement, the limited CNS penetration of these agents, selection of biologically aggressive disease, and the high-risk nature of relapsed/refractory populations rather than a direct causal effect of immunotherapy itself. These findings underscore the need for concurrent intrathecal prophylaxis with all regimens that incorporate these agents [5].

### Blinatumomab

Blinatumomab, a bispecific T-cell engager antibody targeting CD3 and CD19, has transformed both frontline and salvage regimens in B-ALL. However, as a relatively large bispecific antibody (~ 54 kDa), it has limited CNS penetration, and patients with clinically relevant CNS pathology were excluded from pivotal studies [38]. Unfortunately, the CNS has emerged as an important site of relapse during blinatumomab-containing regimens, particularly among heavily pretreated patients [39]. Among 89 patients with blinatumomab failure, 43% relapsed at extramedullary sites, with CNS failure occurring in 39% of those with extramedullary disease (EMD) [40]. Thus, the FDA prescribing information explicitly recommends intrathecal chemotherapy prophylaxis before and during blinatumomab therapy.

### Inotuzumab Ozogamicin

**Fig. 1:**
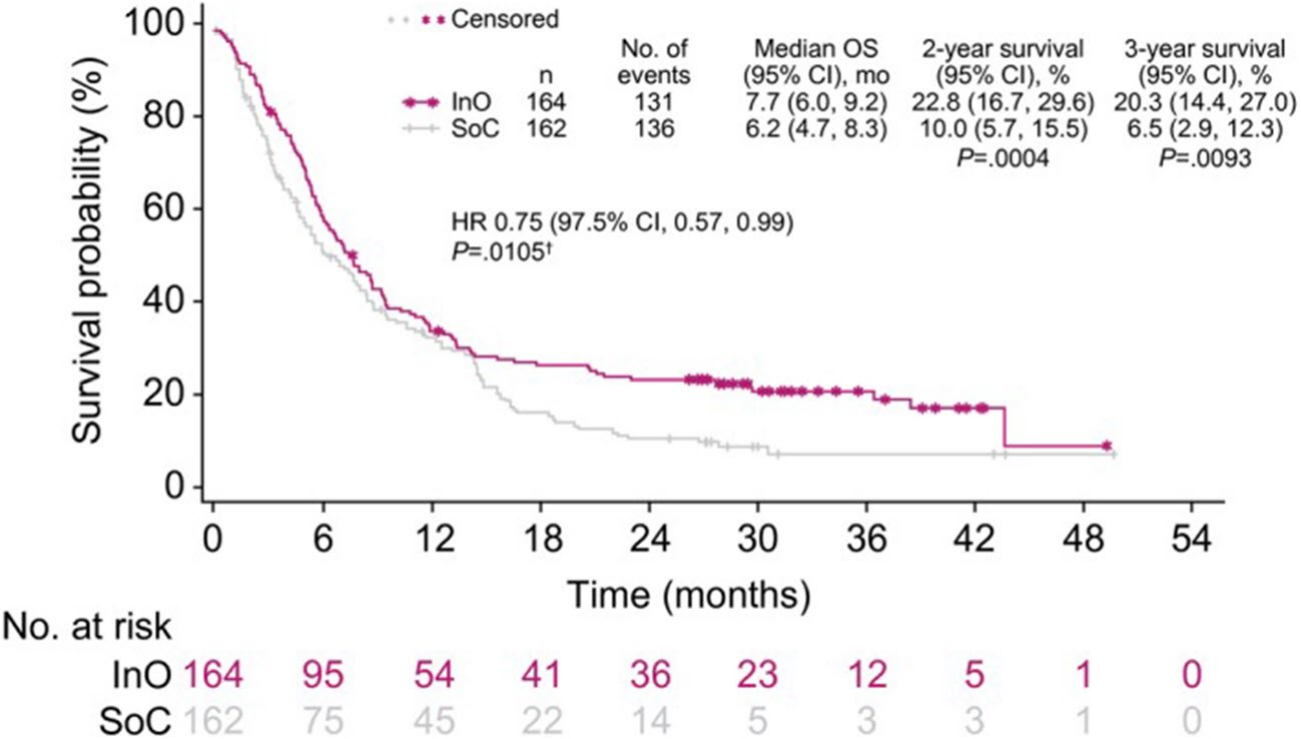
Overall survival in the INO-VATE phase 3 trial. Kaplan–Meier curves for overall survival in patients with relapsed/refractory CD22-positive B-ALL treated with inotuzumab ozogamicin (InO; *n* = 164) versus standard-of-care chemotherapy (SoC; *n* = 162). Two-year OS was 22.8% with InO versus 10.0% with SoC (HR 0.75; *p* = 0.0105). Figure reproduced with permission from Kantarjian, Hagop M et al. “Inotuzumab ozogamicin versus standard of care in relapsed or refractory acute lymphoblastic leukemia: Final report and long-term survival follow-up from the randomized, phase 3 INO-VATE study.” Cancer vol. 125,14 (2019): 2474–2487. doi:10.1002/cncr.32116

Inotuzumab ozogamicin, a CD22-directed antibody-drug conjugate, also has minimal CNS penetration due to its large molecular size (> 150 kDa) [28]. Consequently, patients with active CNS leukemia were excluded from the pivotal INO-VATE trial, a phase 3 randomized study that demonstrated 2-year OS rates of 22.8% versus 10.0% with standard chemotherapy (HR 0.75; *p* = 0.0105), leading to FDA approval of inotuzumab ozogamicin for relapsed/refractory CD22 + B-ALL (Fig. 1) [41, 42]. Despite its systemic efficacy, the inability of inotuzumab ozogamicin to penetrate the CNS has important clinical implications. When combined with mini-hyperfractionated cyclophosphamide, vincristine, and dexamethasone (mini-hyper-CVD) ± blinatumomab in the relapsed/refractory setting, 42% of relapses involved the CNS [43]. In a phase II study of hyper-CVAD with sequential blinatumomab and inotuzumab ozogamicin, 3/3 relapses in the inotuzumab cohort were CNS-only relapses, illustrating that the CNS may remain a sanctuary site despite excellent systemic disease control [44]. Ultimately, certain authors emphasize that the primary role of inotuzumab ozogamicin in the context of CNS disease is as a bridge to allogeneic HSCT or CAR T-cell therapy rather than as a direct CNS treatment [41].

### CAR T-Cell Therapy

CD19-directed CAR T-cell therapy has emerged as a promising modality for CNS leukemia in relapsed/refractory B-ALL, as it is uniquely capable of crossing the BBB. In a landmark study of 48 patients with relapsed/refractory B-ALL and CNS leukemia, CD19-specific CAR T-cell therapy achieved a CNS remission rate of 85.4%, with a 12-month cumulative incidence of CNS relapse significantly lower than the bone marrow relapse rate, suggesting effective CAR T-cell trafficking and persistence within the CNS compartment [45]. Regarding safety, grade 3–4 neurotoxic events occurred in approximately 23% of patients with CNS disease and were associated with higher pre-infusion CNS disease burden [45].

**Fig. 2:**
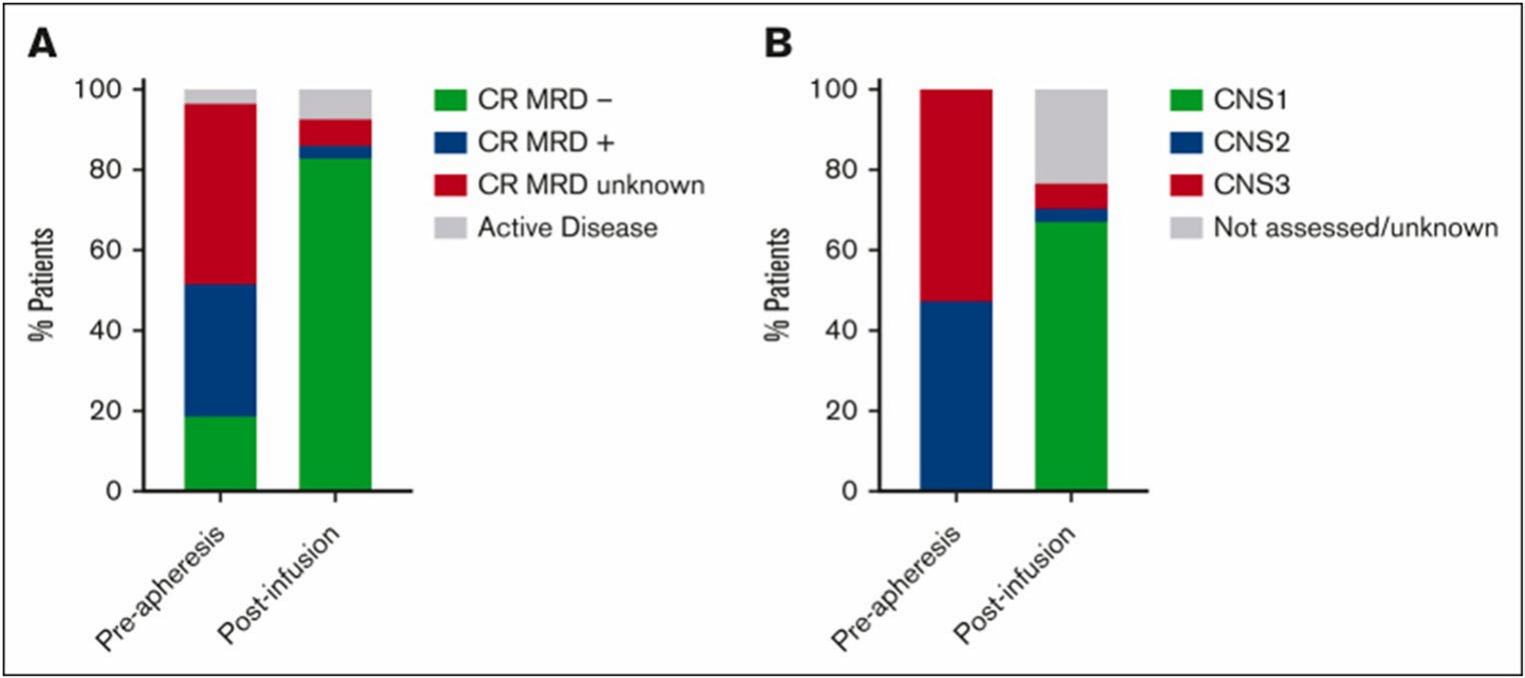
Response rates at pre-apheresis assessment and day + 28 after infusion. **A** Medullary response rates are shown. A total of 28 patients achieved CR, of whom 25 achieved CR with MRD-negative status. Of 13 patients with active disease at pre-apheresis, 11 achieved MRD-negative CR. **B** CNS responses are shown; 21 of 24 evaluable patients achieved a response, whereas 3 patients had refractory disease (1 with CNS-2 disease and 2 with CNS-3 disease). Figure reproduced with permission from Muhsen, Ibrahim N et al. “Outcomes of brexucabtagene autoleucel in patients with relapsed/refractory acute lymphoblastic leukemia with CNS involvement.” Blood advances vol. 9,16 (2025): 4081–4089. doi:10.1182/bloodadvances.2024015779

Among commercially available products, brexucabtagene autoleucel (brexu-cel) is the best studied in this setting. The ZUMA-3 trial established its efficacy in adult relapsed/refractory B-ALL, though it excluded patients with advanced or symptomatic CNS involvement [46, 47]. A subsequent multicenter real-world analysis from the ROCCA (Real-World Outcomes Collaborative for CAR T in ALL) consortium specifically evaluated brexu-cel in CNS B-ALL [48]. Among 189 infused patients, 31 had CNS-2 or CNS-3 disease prior to apheresis; following brexu-cel, 87.5% achieved CNS-1 status, and the majority attained marrow CR with MRD negativity (Fig. 2). Importantly, no statistically significant differences in PFS or OS were observed between patients with and without CNS involvement, and grade 3–4 immune effector cell-associated neurotoxicity syndrome (ICANS) rates were comparable (35.5% vs. 30%) [48].

### Tyrosine Kinase Inhibitors in Philadelphia Chromosome-Positive ALL

TKI selection in Ph + ALL has critical implications for CNS disease control, as available agents differ substantially in BBB penetration [49]. With improvements in systemic disease control, isolated CNS relapse has been reported in 10%–15% of Ph + ALL patients, underscoring the importance of CNS-directed considerations in TKI selection [17, 50]. Imatinib was the first TKI incorporated into ALL treatment regimens, and while its addition to standard chemotherapy was associated with CR rates exceeding 90%, treatment failure due to CNS relapse was observed due to its poor CNS penetration [51]. The NCCN guidelines now recommend that imatinib use in first-line Ph + ALL should be restricted to patients who cannot tolerate broader-acting TKIs [5]. Dasatinib, a second-generation TKI, is characterized by enhanced CNS penetration and has been widely adopted in adult Ph+ ALL [52]. A propensity score-matched analysis of pooled data from three prospective trials comparing dasatinib with imatinib demonstrated significantly higher 3-year EFS (73% vs. 49%; *p* = 0.01) and 3-year OS (85% vs. 60%; *p* = 0.004) with dasatinib [53]. CNS relapse can still occur during dasatinib therapy, attributed to resistant BCR::ABL1 kinase domain mutations.

Ponatinib, a third-generation TKI developed to address resistance mutations, demonstrated higher rates of MRD-negative complete remission compared with imatinib in the PhALLCON trial (34% vs. 17%; *p* = 0.002), leading to regulatory approval as front-line therapy in March 2024 [54]. While data on ponatinib’s CNS pharmacokinetics are more limited than for dasatinib, available evidence suggests ponatinib may have CNS activity, though the extent of penetration and clinical relevance is still unknown [55].

Newer on the market is asciminib, a novel allosteric BCR::ABL1 inhibitor with limited CNS penetration [56, 57]. Combination strategies with CNS-penetrant TKIs, such as dasatinib, are under investigation, and asciminib plus dasatinib has been incorporated into the NCCN guidelines as an “other recommended” regimen for relapsed/refractory Ph + B-ALL [5, 58, 59]. Clinical evidence evaluating asciminib in patients with CNS involvement remains limited, and further studies incorporating CNS-specific endpoints will be essential to define its role in this high-risk population.

## Investigational Approaches

Beyond the established agents discussed above, several investigational strategies are under active exploration to improve CNS disease control in adult ALL, spanning next-generation CAR T-cell platforms, inhibition of leukemic cell trafficking pathways (α6 integrin), anti-vascular endothelial growth factor (VEGF) strategies, and interleukin-15 (IL-15) signaling modulation. Early preclinical and phase I studies suggest these approaches may enhance CNS disease control, although clinical validation is needed.

**Fig. 3:**
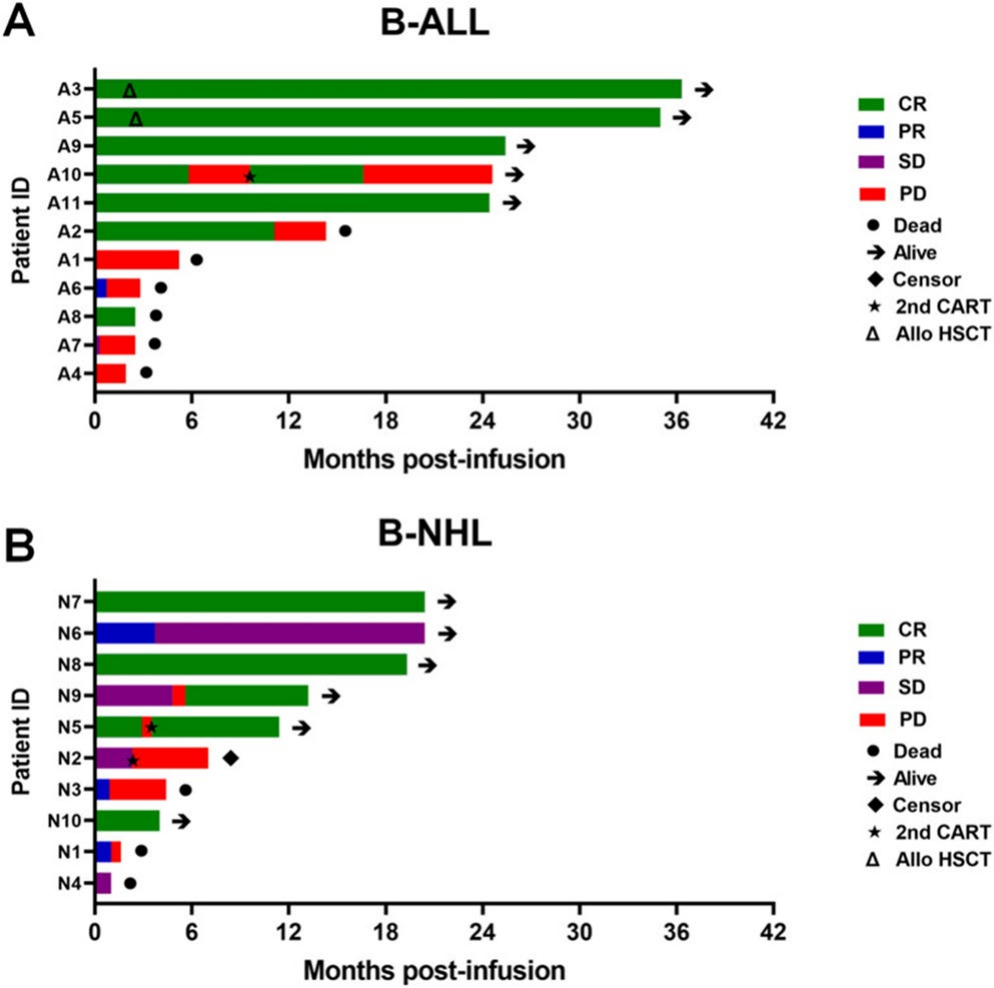
Clinical responses of patients with CNS involvement following 1928zT2 CAR T-cell infusion. **A**, **B**. Swimmer plots showing the clinical responses and follow-up of individual patients treated with 1928zT2 CAR T-cells, as indicated with different colors in the swimmer lanes. Each bar represents one patient. Patients A3 and A5 experienced complete remission and underwent allo-HSCT afterwards. Patients A10, N2 and N5 received a second 1928zT2 CAR T-cell infusion following disease progression. Figure reproduced with permission from He, Bailin et al. “Efficacy and safety of third-generation CD19-CAR T cells incorporating CD28 and TLR2 intracellular domains for B-cell malignancies with central nervous system involvement: results of a pivotal trial.” Journal of translational medicine vol. 23,1 594. 27 May. 2025, doi:10.1186/s12967-025-06608-x

Next-generation CAR T-cell constructs aim to enhance persistence, efficacy, and CNS penetration beyond current CD19-directed therapies. Memory-enriched CD19-directed CAR T-cells derived from naïve, stem, and central memory (Tn/mem) subsets have shown promising activity in a phase I/II study of 46 adults with relapsed/refractory B-ALL, where 87% achieved CR/CRi [60]. Notably, 35% had extramedullary disease at lymphodepletion, and 13 of 15 evaluable patients with EMD responded, including those with CNS involvement, with a favorable safety profile (grade ≥ 3 CRS 7%; grade ≥ 3 neurotoxicity 17%). Additionally, third-generation constructs incorporating CD28 and TLR2 costimulatory domains demonstrated a 73% overall response rate in 11 patients with relapsed/refractory B-ALL and CNS involvement (grade ≥ 3 CRS 14%; grade ≥ 3 ICANS 28.6%), with CSF detection of CAR T-cells associated with significantly improved outcomes [61]. Figure 3 showcases swimmer plot analysis of patients with B-ALL and B-cell Non-Hodgkin Lymphoma (B-NHL), highlighting durable complete responses and prolonged survival in a subset of patients. Future directions include prophylactic CAR T-cell consolidation for patients at high risk of CNS relapse and dual-targeting constructs (CD19/CD22) to address antigen escape, a key mechanism underlying relapse after CD19-directed therapy [62].

Preclinical work has also identified promising non-cellular targets. Yao et al. demonstrated that ALL cells do not enter the BBB directly but instead migrate into the CNS along bridging vessels that link the subarachnoid space and the vertebral/calvarial bone marrow, mediated by the laminin receptor α6 integrin [63]. Treatment with anti-α6 integrin-neutralizing antibodies or PI3Kδ inhibitors (which decrease α6 integrin expression) significantly reduced CSF blast counts in xenografted mice, and in vivo α6 deletion combined with a TKI outperformed TKI therapy alone, supporting α6 as a dual target for chemoresistance and CNS prophylaxis [63, 64]. Similarly, VEGF has also been identified as a mediator of ALL cell entry into the CNS [65]. Bevacizumab (a VEGF inhibitor) decreased CNS leukemia in a murine model, suggesting anti-VEGF strategies as a potential novel approach to mitigate CNS disease [65]. Finally, IL-15 signaling modulation has shown early clinical promise: a phase 1 trial combining a novel bispecific CD19/CD22 CAR with an IL-15 receptor agonist yielded 10-fold increases in CSF CAR T-cell concentrations, MRD-negative remission in 89% of patients, and significantly improved 12-month PFS compared to historical controls (67% vs. 38%) [66]. Further studies are necessary to transform these investigational strategies into clinical practice and improve CNS disease control in adult ALL.

## Special Populations and Considerations

### T-Cell ALL

T-cell ALL presents unique challenges for CNS disease management [67]. T-ALL is an established risk factor for CNS relapse and carries worse outcomes than B-ALL, with a lower five-year event-free survival (80% vs. 90%) [68]. The therapeutic backbone relies on higher doses of methotrexate, cytarabine, and asparaginase, with the addition of nelarabine, a nucleoside analog prodrug with selective toxicity against T-lymphoblasts [68]. Critically, T-ALL lacks the CD19, CD20, and CD22 surface antigens targeted by blinatumomab, inotuzumab ozogamicin, and currently approved CAR T-cell products, limiting the applicability of these immunotherapeutic agents for both systemic and CNS disease control [19].

Among emerging treatment strategies, venetoclax combined with hyper-CVAD, nelarabine, and pegylated asparaginase was evaluated in a phase 2 trial of 145 adults with T-ALL and T-cell lymphoblastic lymphoma (T-LBL), demonstrating a 5-year OS of 66% for the overall cohort; the venetoclax-containing sub-cohort showed a superior 2-year PFS of 88% versus 64% (*p* = 0.03) compared to the original cohort without venetoclax [69]. In a separate phase 1b study of venetoclax plus mini-hyper-CVD in older adults (≥ 60 years) with newly diagnosed ALL (including T-ALL), 10 of 11 patients (91%) achieved MRD-negative CR, with a median disease-free survival of 54.6 months [70]. CD7-directed CAR T-cells have also shown promise in relapsed/refractory T-ALL. In the largest reported series of 60 patients, the day-28 bone marrow CR rate was 94%, and the 2-year OS was 64% [71]. A phase 1 study of universal base-edited anti-CD7 CAR T cells (BE-CAR7) reported morphologic remission in all 11 treated patients (however, only 2 adults), with 82% achieving deep remission sufficient to proceed to allogeneic transplant [72]. As these novel agents are integrated into T-ALL treatment, the adequacy of CNS prophylaxis must be carefully maintained, given the absence of CNS-penetrating immunotherapeutic options comparable to those available in B-ALL.

### Older Adults

Older adults (≥ 65 years) with ALL represent a particularly vulnerable population for whom CNS-directed therapy poses distinct challenges [73]. Many patients in this age group cannot tolerate intensive chemotherapy, and the toxicities of intrathecal chemotherapy and cranial irradiation can be prohibitive [19]. However, the NCCN guidelines emphasize that chronological age is a poor surrogate for fitness and recommend individualized assessment based on performance status and comorbidities [5]. Dose reductions of pegaspargase (1,000 IU/m²), anthracyclines (50% dose), and/or other myelosuppressive agents may be warranted, but all regimens should include CNS prophylaxis, antimicrobial prophylaxis, and growth factor support [5].

Lower-intensity regimens incorporating inotuzumab ozogamicin and blinatumomab have shown favorable results in older patients; the mini-hyper-CVD regimen with inotuzumab and sequential blinatumomab demonstrated 5-year PFS of 44% among 80 patients (median age 68 years), and the Alliance A041703 chemotherapy-free regimen of inotuzumab followed by blinatumomab yielded a composite CR rate of 97% and 1-year EFS of 75% in 33 patients (median age 71 years) [43, 74]. However, the limited CNS penetration of these agents necessitates more frequent intrathecal chemotherapy to mitigate CNS relapse risk. This creates a particular problem in older adults, as the cumulative neurotoxicity of intrathecal chemotherapy has been associated with white matter microstructural changes and cognitive impairment, and older patients are especially susceptible to methotrexate-induced cognitive dysfunction, which can manifest as delirium, posterior reversible encephalopathy syndrome (PRES), or leukoencephalopathy [75, 76]. Balancing adequate CNS prophylaxis against the heightened risk of neurotoxicity requires individualized treatment planning and close neurologic monitoring.

## Institutional Approach to B-ALL Treatment and CNS-Directed Therapy

At our institution, the approach to systemic therapy for B-ALL is stratified by Philadelphia chromosome status, patient age, and fitness. For Ph-negative B-ALL, young and fit patients are preferentially treated with the CALGB 10403 pediatric-inspired regimen, which is the most frequently used treatment for young adults in the United States [77]. For patients treated in the community setting where the logistical demands of CALGB 10403 may be prohibitive, HyperCVAD is considered an alternative. Older or less fit patients are treated with E1910, HyperCVAD, or inotuzumab/blinatumomab-based regimens, consistent with NCCN-recommended options for adults ≥ 65 years or those with substantial comorbidities [5]. Regardless of the backbone regimen chosen, blinatumomab is incorporated into consolidation and/or MRD-negative post-remission therapy for all eligible patients, extrapolated from the E1910 data demonstrating a significant overall survival benefit with the addition of blinatumomab (3-year OS 85% vs. 69%; HR 0.42; *P* = 0.003) [38]. For Ph-positive B-ALL, the preferred approach is a TKI (dasatinib or ponatinib) combined with blinatumomab, reflecting the growing body of evidence supporting chemotherapy-free regimens in this population. Alternatively, a TKI may be added to one of the Ph-negative backbone regimens (e.g., HyperCVAD + TKI) when a chemotherapy-containing approach is preferred.

### CNS-Directed Therapy

At our institution, CNS-directed therapy in adult B-ALL is individualized based on CNS status at diagnosis, overall risk stratification, and the systemic treatment regimen employed. For patients with CNS-positive disease (CNS-3), the preferred IT regimen is TIT with cytarabine, methotrexate, and hydrocortisone, administered per the standard intensified schedule of twice-weekly dosing until CSF clearance, followed by a tapering strategy. For patients who cannot tolerate TIT, an alternating single-agent approach is used, cycling between cytarabine ± hydrocortisone and methotrexate ± hydrocortisone. For CNS-negative patients (CNS-1/CNS-2), the IT regimen and schedule are guided by the systemic treatment protocol. When patients are enrolled on a protocol with defined IT recommendations (such as CALGB 10403, E1910, or HyperCVAD), the protocol-specified IT schedule is followed. For patients receiving inotuzumab and/or blinatumomab, agents with limited CNS penetration, our preferred approach is alternating IT cytarabine and IT methotrexate, consistent with the approach used in the hyper-CVAD plus blinatumomab studies, which employed 8 doses of IT methotrexate 12 mg alternating with IT cytarabine 100 mg [78]. If patients develop symptoms following lumbar puncture with IT therapy, hydrocortisone is added to the IT regimen.

The total number of IT doses is determined by risk stratification and treatment context. For high-risk patients, a minimum of 15 IT doses is targeted. For patients proceeding to allogeneic HSCT, at least 8 IT doses are administered prior to transplant, while all other patients are treated with a goal of 12–15 total IT doses. When an established protocol specifies the number and frequency of IT doses, that protocol is followed.

A special consideration applies to Ph-positive patients presenting with hyperleukocytosis who are planned for a chemotherapy-free regimen with blinatumomab. In these patients, at least some systemic CNS-penetrating chemotherapy is administered, specifically, high-dose methotrexate and high-dose cytarabine (HyperCVAD module B), to mitigate the elevated risk of CNS relapse.

## Conclusion

CNS involvement in adult ALL remains a formidable clinical challenge. Although advances in intrathecal therapy and CNS-penetrating systemic chemotherapy have substantially reduced CNS relapse, this progress is challenged in the era of immunotherapy. Agents such as blinatumomab and inotuzumab ozogamicin improve systemic disease control but have limited CNS penetration, underscoring the continued vulnerability of the CNS and the ongoing need for effective intrathecal prophylaxis. CAR T-cell therapy has emerged as a promising modality with the ability to overcome the BBB and induce meaningful responses in CNS disease. Nevertheless, CNS relapse continues to be associated with poor outcomes, highlighting the need for improved preventive strategies, earlier detection, and more effective CNS-directed therapies. Future efforts should focus on optimizing integration of immunotherapy with CNS prophylaxis, refining risk stratification for CNS relapse, and advancing novel approaches targeting leukemic trafficking and persistence within the CNS. Ultimately, as the therapeutic landscape of adult ALL continues to evolve, the integration of CNS-directed strategies into every treatment algorithm will be essential to translating systemic disease control into durable, long-term remission.

## Key References

- Del Principe MI, Maurillo L, Buccisano F, et al. Central nervous system involvement in adult acute lymphoblastic leukemia: diagnostic tools, prophylaxis, and therapy. Mediterr J Hematol Infect Dis. 2014;6(1):e2014075. Published 2014 Nov 1. 10.4084/MJHID.2014.075.
- Acute Lymphoblastic Leukemia. National Comprehensive Cancer Network. Updated 2025-06-27.
- Kopmar NE, Cassaday RD. How I prevent and treat central nervous system disease in adults with acute lymphoblastic leukemia. Blood. 2023;141(12):1379–1388. 10.1182/blood.2022017035.

These key references were picked for their influence on the content and conclusions of this review. These works were among the most frequently cited throughout the manuscript and were selected because they represent pivotal contributions to the field.

## Data Availability

No datasets were generated or analysed during the current study.

## References

[1] Tevatia MS, Sharma I, Jadhav T, Somasundaram V, Sharma S. Isolated CNS Relapse in Acute Lymphoblastic Leukemia (ALL): An Experience from a Tertiary Care Center. J Lab Physicians. 2021;13(2):134–8. 10.1055/s-0041-1730752. 34483558 10.1055/s-0041-1730752 PMC8409115

[2] Liu S, Wang Y. Diagnosis and management of adult central nervous system leukemia. Blood Sci. 2023;5(3):141–9. 10.1097/BS9.0000000000000162. Published 2023 May 30. 37546706 10.1097/BS9.0000000000000162 PMC10400053

[3] Del Principe MI, Maurillo L, Buccisano F, et al. Central nervous system involvement in adult acute lymphoblastic leukemia: diagnostic tools, prophylaxis, and therapy. Mediterr J Hematol Infect Dis. 2014;6(1):e2014075. 10.4084/MJHID.2014.075. Published 2014 Nov 1. 10.4084/MJHID.2014.075 PMC4235468 25408861

[4] Hu Z. E S. Challenges and Advances in the Detection of Leukemic Blasts in Cerebrospinal Fluid in Pediatric Acute Lymphoblastic Leukemia. Cancers (Basel). 2026;18(5):840. Published 2026 Mar 5. 10.3390/cancers18050840 10.3390/cancers18050840 PMC12984855 41827773

[5] Acute Lymphoblastic Leukemia. National Comprehensive Cancer Network. Updated 2025-06-27.

[6] Hasbun R, van de Beek D, Brouwer MC, Bewley J, Bhimraj A, Bhatt DL, et al. Consensus guidelines for lumbar puncture in patients with neurological diseases. Clin Infect Dis. 2021;73(6):e1903–14. 10.1093/cid/ciaa777.

[7] Guenette JP, Tirumani SH, Keraliya AR, Shinagare AB, Ramaiya NH, Jagannathan JP. MRI Findings in Patients With Leukemia and Positive CSF Cytology: A Single-Institution 5-Year Experience. AJR Am J Roentgenol. 2016;207(6):1278–82. 10.2214/AJR.16.16221. 27611654 10.2214/AJR.16.16221

[8] Freilich RJ, Krol G, DeAngelis LM. Neuroimaging and cerebrospinal fluid cytology in the diagnosis of leptomeningeal metastasis. Ann Neurol. 1995;38(1):51–7. 10.1002/ana.410380111. 7611725 10.1002/ana.410380111

[9] Winick N, Devidas M, Chen S, et al. Impact of Initial CSF Findings on Outcome Among Patients With National Cancer Institute Standard- and High-Risk B-Cell Acute Lymphoblastic Leukemia: A Report From the Children’s Oncology Group. J Clin Oncol. 2017;35(22):2527–34. 10.1200/JCO.2016.71.4774. 28535084 10.1200/JCO.2016.71.4774 PMC5536164

[10] Barranco-Lampón G, Rozen-Fuller E, Olarte-Carrilo I, Martínez-Tovar A, León-González G, Castellanos-Sinco H, et al. Association between traumatic lumbar puncture and the risk of central nervous system relapse in adults with acute lymphoblastic leukaemia. Rev Med Hosp Gen Mex. 2015;78(3):124–8. 10.1016/j.hgmx.2015.05.004.

[11] Mitri Z, Siddiqui MT, El Rassi F, et al. Sensitivity and specificity of cerebrospinal fluid flow cytometry for the diagnosis of leukemic meningitis in acute lymphoblastic leukemia/lymphoma. Leuk Lymphoma. 2014;55(7):1498–500. 10.3109/10428194.2013.852667. 24134778 10.3109/10428194.2013.852667

[12] Del Principe MI, Buzzatti E, Piciocchi A, et al. Clinical significance of occult central nervous system disease in adult acute lymphoblastic leukemia. A multicenter report from the Campus ALL Network. Haematologica. 2021;106(1):39–45. 10.3324/haematol.2019.231704. Published 2021 Jan 1. 31879328 10.3324/haematol.2019.231704 PMC7776237

[13] Dargenio M, Bonifacio M, Chiaretti S, et al. Incidence, treatment and outcome of central nervous system relapse in adult acute lymphoblastic leukaemia patients treated front-line with paediatric-inspired regimens: A retrospective multicentre Campus ALL study. Br J Haematol. 2023;200(4):440–50. 10.1111/bjh.18537. 36335916 10.1111/bjh.18537 PMC10098932

[14] Gökbuget N. Treatment of older patients with acute lymphoblastic leukemia. Hematol Am Soc Hematol Educ Program. 2016;2016(1):573–9. 10.1182/asheducation-2016.1.573. 10.1182/asheducation-2016.1.573 PMC6142461 27913531

[15] Sancho JM, Ribera JM, Oriol A, et al. Central nervous system recurrence in adult patients with acute lymphoblastic leukemia: frequency and prognosis in 467 patients without cranial irradiation for prophylaxis. Cancer. 2006;106(12):2540–6. 10.1002/cncr.21948. 16700036 10.1002/cncr.21948

[16] Gökbuget N, Boissel N, Chiaretti S, et al. Diagnosis, prognostic factors, and assessment of ALL in adults: 2024 ELN recommendations from a European expert panel. Blood. 2024;143(19):1891–902. 10.1182/blood.2023020794. 38295337 10.1182/blood.2023020794

[17] Liu-Dumlao T, Kantarjian H, Thomas DA, O’Brien S, Ravandi F. Philadelphia-positive acute lymphoblastic leukemia: current treatment options. Curr Oncol Rep. 2012;14(5):387–94. 10.1007/s11912-012-0247-7. 22669492 10.1007/s11912-012-0247-7 PMC4199301

[18] Tasian SK, Loh ML, Hunger SP. Philadelphia chromosome-like acute lymphoblastic leukemia. Blood. 2017;130(19):2064–72. 10.1182/blood-2017-06-743252. 28972016 10.1182/blood-2017-06-743252 PMC5680607

[19] Kantarjian H, Pui CH, Jabbour E. Acute lymphocytic leukaemia. Lancet. 2025;406(10506):950–62. 10.1016/S0140-6736(25)00864-5. 40759141 10.1016/S0140-6736(25)00864-5

[20] ASH Clinical Practice Guidelines on Acute Lymphoblastic Leukemia in Adolescents and Young Adults. American Society of Hematology. (2024). 2024.

[21] Bassan R, Masciulli A, Intermesoli T, et al. Randomized trial of radiation-free central nervous system prophylaxis comparing intrathecal triple therapy with liposomal cytarabine in acute lymphoblastic leukemia. Haematologica. 2015;100(6):786–93. 10.3324/haematol.2014.123273. 25749825 10.3324/haematol.2014.123273 PMC4450624

[22] Jabbour E, O’Brien S, Kantarjian H, et al. Neurologic complications associated with intrathecal liposomal cytarabine given prophylactically in combination with high-dose methotrexate and cytarabine to patients with acute lymphocytic leukemia. Blood. 2007;109(8):3214–8. 10.1182/blood-2006-08-043646. 17209054 10.1182/blood-2006-08-043646

[23] Balis FM, Lester CM, Chrousos GP, Heideman RL, Poplack DG. Differences in cerebrospinal fluid penetration of corticosteroids: possible relationship to the prevention of meningeal leukemia. J Clin Oncol. 1987;5(2):202–7. 10.1200/JCO.1987.5.2.202. 3806166 10.1200/JCO.1987.5.2.202

[24] Mitchell CD, Richards SM, Kinsey SE, et al. Benefit of dexamethasone compared with prednisolone for childhood acute lymphoblastic leukaemia: results of the UK Medical Research Council ALL97 randomized trial. Br J Haematol. 2005;129(6):734–45. 10.1111/j.1365-2141.2005.05509.x. 15952999 10.1111/j.1365-2141.2005.05509.x

[25] Labar B, Suciu S, Willemze R, et al. Dexamethasone compared to prednisolone for adults with acute lymphoblastic leukemia or lymphoblastic lymphoma: final results of the ALL-4 randomized, phase III trial of the EORTC Leukemia Group. Haematologica. 2010;95(9):1489–95. 10.3324/haematol.2009.018580. 20378563 10.3324/haematol.2009.018580 PMC2930949

[26] Olmos-Jiménez R, Espuny-Miró A, Cárceles Rodríguez C, Díaz-Carrasco MS. Practical aspects of the use of intrathecal chemotherapy. Aspectos prácticos de la utilización de quimioterapia intratecal. Farm Hosp. 2017;41(n01):105–29. 10.7399/fh.2017.41.1.10616. Published 2017 Jan 1. 28045655 10.7399/fh.2017.41.1.10616

[27] Van Trimpont M, Peeters E, De Visser Y, et al. Novel Insights on the Use of L-Asparaginase as an Efficient and Safe Anti-Cancer Therapy. Cancers (Basel). 2022;14(4):902. 10.3390/cancers14040902. Published 2022 Feb 11. 35205650 10.3390/cancers14040902 PMC8870365

[28] Kopmar NE, Cassaday RD. How I prevent and treat central nervous system disease in adults with acute lymphoblastic leukemia. Blood. 2023;141(12):1379–88. 10.1182/blood.2022017035. 36548957 10.1182/blood.2022017035 PMC10082377

[29] Jabbour E, Thomas D, Cortes J, Kantarjian H, O’Brien S. Central Nervous System Prophylaxis in Adults with Acute Lymphoblastic Leukemia. Cancer. 2010. 10.1002/cncr.25008 20209620

[30] Garcia-Manero G, Kantarjian HM, Schiffer CA et al. Central Nervous System Prophylaxis and Therapy. In: Kufe DW, Pollock RE, Weichselbaum RR, editors. Holland-F1rei Cancer Medicine. 6th edition. Hamilton (ON): BC Decker; 2003.

[31] Thomas DA, O’Brien S, Rytting M, Ravandi F, Jabbour E, Ferrajoli A, et al. Incidence of central nervous system (CNS) relapse in de novo adult acute lymphoblastic leukemia (ALL). Blood. 2014 Dec;6(21):940–940. [cited 2022 Oct 1].

[32] Reman O, Pigneux A, Huguet F, et al. Central nervous system involvement in adult acute lymphoblastic leukemia at diagnosis and/or at first relapse: results from the GET-LALA group. Leuk Res. 2008;32(11):1741–50. 10.1016/j.leukres.2008.04.011. 18508120 10.1016/j.leukres.2008.04.011

[33] Wu SY, Short NJ, Nasr L, Dabaja BS, Fang PQ. Central Nervous System Prophylaxis and Treatment in Acute Leukemias. Curr Treat Options Oncol. 2022;23(12):1829–44. 10.1007/s11864-022-01032-5. 36510037 10.1007/s11864-022-01032-5 PMC9767998

[34] Pagliuca S, Schmid C, Santoro N, et al. Donor lymphocyte infusion after allogeneic hematopoietic cell transplantation for haematological malignancies: basic considerations and best practice recommendations from the EBMT. Lancet Haematol. 2024;11(6):e448–58. 10.1016/S2352-3026(24)00098-X. 38796194 10.1016/S2352-3026(24)00098-X

[35] Kwag D, Yoon JH, Min GJ, et al. Outcome of second allogeneic hematopoietic cell transplantation in adult patients with relapsed B-cell acute lymphoblastic leukemia in the era of new immunotherapeutic agents. Bone Marrow Transpl. 2025;60(9):1249–57. 10.1038/s41409-025-02639-6. 10.1038/s41409-025-02639-6 40527981

[36] Nagler A, Labopin M, Dholaria B, et al. Second allogeneic stem cell transplantation in patients with acute lymphoblastic leukaemia: a study on behalf of the Acute Leukaemia Working Party of the European Society for Blood and Marrow Transplantation. Br J Haematol. 2019;186(5):767–76. 10.1111/bjh.15973. 31115916 10.1111/bjh.15973

[37] Cassaday RD. Sanctuary sites and extramedullary relapses in the chemo-free world: insights from immunotherapies in B-ALL. Hematol Am Soc Hematol Educ Program. 2025;2025(1):245–51. 10.1182/hematology.2025000711. 10.1182/hematology.2025000711 PMC12891342 41347976

[38] BLINCYTO. FDA Drug Label. Food and Drug Administration. Updated date: 2025-10-21.

[39] Wilke AC, Gökbuget N. The role of blinatumomab in adult acute B lymphoblastic leukaemia. Br J Haematol. 2025;207(1):27–42. 10.1111/bjh.20134. 40368871 10.1111/bjh.20134 PMC12234269

[40] Aldoss I, Otoukesh S, Zhang J, et al. Extramedullary disease relapse and progression after blinatumomab therapy for treatment of acute lymphoblastic leukemia. Cancer. 2022;128(3):529–35. 10.1002/cncr.33967. 34633671 10.1002/cncr.33967

[41] Kantarjian HM, DeAngelo DJ, Stelljes M, et al. Inotuzumab ozogamicin versus standard of care in relapsed or refractory acute lymphoblastic leukemia: Final report and long-term survival follow-up from the randomized, phase 3 INO-VATE study. Cancer. 2019;125(14):2474–87. 10.1002/cncr.32116. 30920645 10.1002/cncr.32116 PMC6618133

[42] Stock W, Martinelli G, Stelljes M, et al. Efficacy of inotuzumab ozogamicin in patients with Philadelphia chromosome-positive relapsed/refractory acute lymphoblastic leukemia. Cancer. 2021;127(6):905–13. 10.1002/cncr.33321. 33231879 10.1002/cncr.33321 PMC7983935

[43] Jabbour E, Short NJ, Senapati J, et al. Mini-hyper-CVD plus inotuzumab ozogamicin, with or without blinatumomab, in the subgroup of older patients with newly diagnosed Philadelphia chromosome-negative B-cell acute lymphocytic leukaemia: long-term results of an open-label phase 2 trial. Lancet Haematol. 2023;10(6):e433–44. 10.1016/S2352-3026(23)00073-X. 37187201 10.1016/S2352-3026(23)00073-X PMC11840755

[44] Short N, Jabbour E, Jain N, et al. P358: Hyper-Cvad With Blinatumomab And Inotuzumab Ozogamicin For Patients With Newly Diagnosed Philadelphia Chromosome-Negative B-Cell Acute Lymphoblastic Leukemia: A Phase Ii Study. Hemasphere. 2023;7(Suppl):e67564ca. 10.1097/01.HS9.0000968344.67564.ca. Published 2023 Aug 8.

[45] Qi Y, Zhao M, Hu Y, et al. Efficacy and safety of CD19-specific CAR T cell-based therapy in B-cell acute lymphoblastic leukemia patients with CNSL. Blood. 2022;139(23):3376–86. 10.1182/blood.2021013733. 35338773 10.1182/blood.2021013733 PMC11022988

[46] Shah BD, Ghobadi A, Oluwole OO, et al. KTE-X19 for relapsed or refractory adult B-cell acute lymphoblastic leukaemia: phase 2 results of the single-arm, open-label, multicentre ZUMA-3 study. Lancet. 2021;398(10299):491–502. 10.1016/S0140-6736(21)01222-8. 34097852 10.1016/S0140-6736(21)01222-8 PMC11613962

[47] Shah BD, Cassaday RD, Park JH, et al. Three-year analysis of adult patients with relapsed or refractory B-cell acute lymphoblastic leukemia treated with brexucabtagene autoleucel in ZUMA-3. Leukemia. 2025;39(5):1058–68. 10.1038/s41375-025-02532-7. 40108332 10.1038/s41375-025-02532-7 PMC12055586

[48] Muhsen IN, Roloff GW, Faramand R, et al. Outcomes of brexucabtagene autoleucel in patients with relapsed/refractory acute lymphoblastic leukemia with CNS involvement. Blood Adv. 2025;9(16):4081–9. 10.1182/bloodadvances.2024015779. 40334068 10.1182/bloodadvances.2024015779 PMC12359224

[49] Secker-Walker LM, Craig JM, Hawkins JM, Hoffbrand AV. Philadelphia positive acute lymphoblastic leukemia in adults: age distribution, BCR breakpoint and prognostic significance. Leukemia. 1991;5:196–9. 2013979

[50] Haddad FG, Jabbour E, Short NJ, Jain N, Kantarjian H. SOHO State of the Art Updates and Next Questions: Update on the Approach to Philadelphia Chromosome-Positive Acute Lymphoblastic Leukemia. Clin Lymphoma Myeloma Leuk. 2024;24(5):271–6. 10.1016/j.clml.2023.12.007. 38185587 10.1016/j.clml.2023.12.007

[51] Haddad FG, Short NJ. Evidence-Based Minireview: What is the optimal tyrosine kinase inhibitor for adults with newly diagnosed Philadelphia chromosome-positive acute lymphoblastic leukemia? Hematol Am Soc Hematol Educ Program. 2022;2022(1):213–7. 10.1182/hematology.2022000413. 10.1182/hematology.2022000413 PMC9820762 36485089

[52] Porkka K, Koskenvesa P, Lundán T, et al. Dasatinib crosses the blood-brain barrier and is an efficient therapy for central nervous system Philadelphia chromosome-positive leukemia. Blood. 2008;112(4):1005–12. 10.1182/blood-2008-02-140665. 18477770 10.1182/blood-2008-02-140665

[53] Nishiwaki S, Sugiura I, Fujisawa S, et al. Dasatinib is superior to imatinib in adult Ph + ALL: a propensity score-matched analysis of pooled JALSG trial data. Int J Hematol. 2026;123(1):24–31. 10.1007/s12185-025-04058-1. 40864405 10.1007/s12185-025-04058-1

[54] Jabbour E, Kantarjian H, Aldoss I, et al. S110: Phallcon: A Phase 3 Study Comparing Ponatinib Versus Imatinib In Newly Diagnosed Ph + All. Hemasphere. 2023;7(Suppl):e68516d0. 10.1097/01.HS9.0000967352.68516.d0. Published 8 Aug 2023.

[55] Ravi K, Franson A, Homan MJ, et al. Comparative pharmacokinetic analysis of the blood-brain barrier penetration of dasatinib and ponatinib in mice. Leuk Lymphoma. 2021;62(8):1990–4. 10.1080/10428194.2021.1894647. 33682631 10.1080/10428194.2021.1894647 PMC8855457

[56] Pamuk GE, Chow ECY, Ionan AC, et al. FDA Approval Summary: Asciminib for Ph + CML in Chronic Phase Treated with Two or More Tyrosine Kinase Inhibitors and for the T315I Mutation. Clin Cancer Res. 2024;30(19):4266–71. 10.1158/1078-0432.CCR-24-1086. 39088257 10.1158/1078-0432.CCR-24-1086 PMC11444873

[57] Lang F, Ottmann OG. Asciminib for Ph + ALL: a step forward? Blood. 2025;145(6):551–2. 10.1182/blood.2024027064. 39913337 10.1182/blood.2024027064

[58] Eide CA, Zabriskie MS, Savage Stevens SL, et al. Combining the Allosteric Inhibitor Asciminib with Ponatinib Suppresses Emergence of and Restores Efficacy against Highly Resistant BCR-ABL1 Mutants. Cancer Cell. 2019;36(4):431–e4435. 10.1016/j.ccell.2019.08.004. 31543464 10.1016/j.ccell.2019.08.004 PMC6893878

[59] Scemblix (asciminib). FDA Drug Label. Food and Drug Administration. Updated date: 2024-10-29.

[60] Aldoss I, Khaled SK, Wang X, et al. Favorable Activity and Safety Profile of Memory-Enriched CD19-Targeted Chimeric Antigen Receptor T-Cell Therapy in Adults with High-Risk Relapsed/Refractory ALL. Clin Cancer Res. 2023;29(4):742–53. 10.1158/1078-0432.CCR-22-2038. 36255386 10.1158/1078-0432.CCR-22-2038 PMC10544259

[61] He B, Lin R, Xu N, et al. Efficacy and safety of third-generation CD19-CAR T cells incorporating CD28 and TLR2 intracellular domains for B-cell malignancies with central nervous system involvement: results of a pivotal trial. J Transl Med. 2025;23(1):594. 10.1186/s12967-025-06608-x. Published 2025 May 27. 40426201 10.1186/s12967-025-06608-x PMC12117732

[62] de Oliveira Canedo G, Roddie C, Amrolia PJ. Dual-targeting CAR T cells for B-cell acute lymphoblastic leukemia and B-cell non-Hodgkin lymphoma. Blood Adv. 2025;9(4):704–21. 10.1182/bloodadvances.2024013586. 39631066 10.1182/bloodadvances.2024013586 PMC11869864

[63] Yao H, Price TT, Cantelli G, et al. Leukaemia hijacks a neural mechanism to invade the central nervous system. Nature. 2018;560(7716):55–60. 10.1038/s41586-018-0342-5. 30022166 10.1038/s41586-018-0342-5 PMC10257142

[64] Gang EJ, Kim HN, Hsieh YT, et al. Integrin α6 mediates the drug resistance of acute lymphoblastic B-cell leukemia. Blood. 2020;136(2):210–23. 10.1182/blood.2019001417. 32219444 10.1182/blood.2019001417 PMC7357190

[65] Münch V, Trentin L, Herzig J, et al. Central nervous system involvement in acute lymphoblastic leukemia is mediated by vascular endothelial growth factor. Blood. 2017;130(5):643–54. 10.1182/blood-2017-03-769315. 28550041 10.1182/blood-2017-03-769315

[66] Srinagesh H, Jackson C, Shiraz P, et al. A phase 1 clinical trial of NKTR-255 with CD19-22 CAR T-cell therapy for refractory B-cell acute lymphoblastic leukemia. Blood. 2024;144(16):1689–98. 10.1182/blood.2024024952. 38968138 10.1182/blood.2024024952 PMC11522888

[67] Raetz EA, Teachey DT. T-cell acute lymphoblastic leukemia. Hematol Am Soc Hematol Educ Program. 2016;2016(1):580–8. 10.1182/asheducation-2016.1.580. 10.1182/asheducation-2016.1.580 PMC6142501 27913532

[68] Hormann FM, Rudd SG. Nelarabine in T-cell acute lymphoblastic leukemia: intracellular metabolism and molecular mode-of-action. Leukemia. 2025;39(3):531–42. 10.1038/s41375-025-02529-2. 39962329 10.1038/s41375-025-02529-2 PMC11879874

[69] Ravandi F, Senapati J, Jain N, et al. Longitudinal follow up of a phase 2 trial of venetoclax added to hyper-CVAD, nelarabine and pegylated asparaginase in patients with T-cell acute lymphoblastic leukemia and lymphoma. Leukemia. 2024;38:2717–21. 10.1038/s41375-024-02414-4. 39322712 10.1038/s41375-024-02414-4

[70] Luskin MR, Shimony S, Keating J, et al. Venetoclax plus low-intensity chemotherapy for adults with acute lymphoblastic leukemia. Blood Adv. 2025;9(3):617–26. 10.1182/bloodadvances.2024014405. 39546748 10.1182/bloodadvances.2024014405 PMC11847096

[71] Zhang X, Yang J, Li J, et al. Analysis of 60 patients with relapsed or refractory T-cell acute lymphoblastic leukemia and T-cell lymphoblastic lymphoma treated with CD7-targeted chimeric antigen receptor-T cell therapy. Am J Hematol. 2023;98(12):1898–908. 10.1002/ajh.27094. 37740926 10.1002/ajh.27094

[72] Chiesa R, Georgiadis C, Rashed H, et al. Universal Base-Edited CAR7 T Cells for T-Cell Acute Lymphoblastic Leukemia. N Engl J Med. 2026;394(2):152–65. 10.1056/NEJMoa2505478. 41363805 10.1056/NEJMoa2505478

[73] Bruzzese A, Vigna E, Martino EA, et al. Ph-Negative Acute Lymphoblastic Leukemia in the Older Adults: Biology, Therapeutic Strategies and Unmet Needs. Eur J Haematol Published online March. 2026;15. 10.1111/ejh.70169. 10.1111/ejh.70169 41833299

[74] Wieduwilt MJ, Yin J, Kour O, et al. Inotuzumab Ozogamicin Then Blinatumomab for Older Adults With Newly Diagnosed B-Cell ALL: Alliance Study A041703 Cohort 1 Results. J Clin Oncol. 2025;43(32):3526–35. 10.1200/JCO-25-00307. 41026957 10.1200/JCO-25-00307 PMC13093227

[75] Laniel J, Sultan S, Sinnett D, et al. Cumulative Dosage of Intrathecal Chemotherapy Agents Predicts White Matter Integrity in Long-Term Survivors of Acute Lymphoblastic Leukemia: A PETALE Study. Cancers (Basel). 2024;16(6):1208. 10.3390/cancers16061208. Published 2024 Mar 19. 38539543 10.3390/cancers16061208 PMC10969288

[76] Khasraw M, Posner JB. Neurological complications of systemic cancer. Lancet Neurol. 2010;9(12):1214–27. 10.1016/S1474-4422(10)70220-9. 21087743 10.1016/S1474-4422(10)70220-9

[77] Tinajero J, Ngo D, Motta M, et al. The integration of blinatumomab consolidation in the CALGB 10403 regimen for adults with B-cell precursor acute lymphoblastic leukaemia in measurable residual disease-negative remission. Br J Haematol. 2025;207(2):426–31. 10.1111/bjh.20200. 40492422 10.1111/bjh.20200

[78] Richard-Carpentier G, Kantarjian HM, Short NJ, Jabbour E. Updated Results from the Phase II Study of Hyper-CVAD in Sequential Combination with Blinatumomab in Newly Diagnosed Adults with B-Cell Acute Lymphoblastic Leukemia (B-ALL). Blood. 2019;134(Supplement1):3807. 10.1182/blood-2019-129657.

